# Prediction of malaria positivity using patients’ demographic and environmental features and clinical symptoms to complement parasitological confirmation before treatment

**DOI:** 10.1186/s40794-023-00208-7

**Published:** 2023-12-15

**Authors:** Taiwo Adetola Ojurongbe, Habeeb Abiodun Afolabi, Kehinde Adekunle Bashiru, Waidi Folorunso Sule, Sunday Babatunde Akinde, Olusola Ojurongbe, Nurudeen A. Adegoke

**Affiliations:** 1 Department of Statistics, Osun State University, Osogbo, Nigeria; 2 Department of Microbiology, Osun State University, Osogbo, Nigeria; 3 Department of Medical Microbiology and Parasitology, Ladoke Akintola University of Technology, Ogbomoso, Nigeria; 4 Center for Emerging and Re-emerging Infectious Diseases, Ladoke Akintola University of Technology, Ogbomoso, Nigeria; 5 Melanoma Institute Australia, The University of Sydney, Sydney, Australia

**Keywords:** Environmental features, Malaria, Machine learning, Prediction, Social-demographical behaviour, Symptoms

## Abstract

**Background:**

Current malaria diagnosis methods that rely on microscopy and Histidine Rich Protein-2 (HRP2)-based rapid diagnostic tests (RDT) have drawbacks that necessitate the development of improved and complementary malaria diagnostic methods to overcome some or all these limitations. Consequently, the addition of automated detection and classification of malaria using laboratory methods can provide patients with more accurate and faster diagnosis. Therefore, this study used a machine-learning model to predict *Plasmodium falciparum* (*Pf*) antigen positivity (presence of malaria) based on sociodemographic behaviour, environment, and clinical features.

**Method:**

Data from 200 Nigerian patients were used to develop predictive models using nested cross-validation and sequential backward feature selection (SBFS), with 80% of the dataset randomly selected for training and optimisation and the remaining 20% for testing the models. Outcomes were classified as Pf-positive or Pf-negative, corresponding to the presence or absence of malaria, respectively.

**Results:**

Among the three machine learning models examined, the penalised logistic regression model had the best area under the receiver operating characteristic curve for the training set (AUC = 84%; 95% confidence interval [CI]: 75–93%) and test set (AUC = 83%; 95% CI: 63–100%). Increased odds of malaria were associated with higher body weight (adjusted odds ratio (AOR) = 4.50, 95% CI: 2.27 to 8.01, p < 0.0001). Even though the association between the odds of having malaria and body temperature was not significant, patients with high body temperature had higher odds of testing positive for *the Pf* antigen than those who did not have high body temperature (AOR = 1.40, 95% CI: 0.99 to 1.91, p = 0.068). In addition, patients who had bushes in their surroundings (AOR = 2.60, 95% CI: 1.30 to 4.66, p = 0.006) or experienced fever (AOR = 2.10, 95% CI: 0.88 to 4.24, p = 0.099), headache (AOR = 2.07; 95% CI: 0.95 to 3.95, p = 0.068), muscle pain (AOR = 1.49; 95% CI: 0.66 to 3.39, p = 0.333), and vomiting (AOR = 2.32; 95% CI: 0.85 to 6.82, p = 0.097) were more likely to experience malaria. In contrast, decreased odds of malaria were associated with age (AOR = 0.62, 95% CI: 0.41 to 0.90, p = 0.012) and BMI (AOR = 0.47, 95% CI: 0.26 to 0.80, p = 0.006).

**Conclusion:**

Newly developed routinely collected baseline sociodemographic, environmental, and clinical features to predict *Pf* antigen positivity may be a valuable tool for clinical decision-making.

## Background

Malaria is a life-threatening disease caused by *Plasmodium* parasite, transmitted to humans through the bites of *Plasmodium*-infected female Anopheles mosquitoes [1]. Different species of *Plasmodium* cause malaria in humans, with *Plasmodium falciparum* (*Pf*) being the most lethal and prevalent in Africa [2]. Other species that cause malaria in humans include *Plasmodium vivax*, *Plasmodium malariae*, *Plasmodium ovale*, and *Plasmodium knowlesi* [2]. One of the most devastating complications associated with *Pf* infection is cerebral malaria [3–5], a severe disease characterised by vascular leakage and cerebral swelling that can lead to coma and death [5–7]. This complication can be difficult to diagnose and treat, significantly contributing to the high malaria mortality rate in sub-Saharan Africa [5, 8].

Malaria is endemic to sub-Saharan Africa [9–11], where 29 countries account for 96% of global malaria cases [12]. Nigeria, in particular, has one of the highest malaria burdens globally and is a significant contributor to the global malaria mortality rate [13]. Approximately 100 million malaria cases are reported annually in Nigeria, resulting in over 300,000 deaths [13]. Along with the Republic of Congo, Nigeria accounts for 36% of global malaria cases [13]. Given the health implications of malaria, Nigeria has joined other African countries to eradicate the disease between 2025 and 2030 [14]. In addition to the Federal Ministry of Health’s National Malaria Elimination Programme (NMEP), the President established the “Nigeria End Malaria Council” in August 2022 to reduce the malaria burden in the country and serve as a platform to solicit funds to promote malaria elimination [3, 15–17]. Several control measures, including the distribution of long-lasting insecticide-treated mosquito nets, provision of malaria chemopreventive drugs, and utilisation of indoor residual insecticide spray, among other strategies to eradicate malaria, have been implemented by various African governments [18–22]. However, despite ongoing efforts by African governments to combat malaria, it remains a significant public health challenge and continues to affect the continent’s population and economy [23].

The World Health Organization (WHO) recommends prompt malaria diagnosis, either by microscopy or rapid diagnostic tests (RDTs), for all suspected malaria cases before treatment [24]. Microscopy is still considered the “gold standard” for malaria diagnosis in endemic countries. This method has a sensitivity of 50–500 parasites [25], is cost-effective, and enables species and parasite density identification [26, 27]. However, multiple fields must be examined to detect infection, which requires the expertise of at least two microscopists [6]. Hence, the diagnostic accuracy of microscopy is often lacking [6]. Other limitations of microscopic diagnosis include a high number of false negatives, shortage of skilled microscopists, inadequate quality control, and possibility of misdiagnosis due to low parasitaemia or mixed infections [28–30].

RDTs are recommended by the WHO as a good alternative to microscopy in remote areas of Sub-Saharan Africa, with histidine-rich protein II (HRP2)-based RDT being the most used. Some studies have shown that RDT is more sensitive than microscopy [31, 32]. However, false positives are a significant limitation of RDTs, because HRP2 remains in the blood for several days after infection clearance. Furthermore, false negatives can occur because of gene deletions, necessitating an improved and complementary approach to overcome some of these shortcomings.

Accurate and prompt diagnosis of malaria is crucial for effective decision-making, better patient care, and illness management. Correctly identifying which patient needs to take malaria drug(s) and should undergo additional examinations will prevent the overuse of malaria medications and significantly reduce deaths attributable to malaria [33, 34]. Numerous studies have demonstrated machine-learning benefits for different healthcare systems [35–38]. Recently, several studies have used supervised learning algorithms to identify malaria [39–42]. However, despite the success of machine learning in managing malaria, most of its applications concentrate on microscopic image analysis to diagnose malaria, while ignoring the fact that most healthcare institutions in the rural areas of most malaria-endemic countries lack basic facilities to make accurate diagnoses.

Given the widespread practice of self-medication with anti-malarial drugs and the difficulties facing Africa’s health system, a machine learning-based diagnosis model is essential. Additionally, for individuals who cannot obtain a laboratory-based diagnosis, the model can help in accurately diagnosing malaria. Machine learning-based diagnostic tools may provide a simple yet reliable method for assessing the potential malaria status. Hence, this study used patient symptoms, demographic and environmental features to develop a clinical tool for prompt and accurate malaria diagnosis.

## Methods

### Study area, design, and participants

Cross-sectional sampling was conducted in Osogbo, the capital of Osun State, Southwest Nigeria, between June and November 2022 (rainy to dry season). In addition, the entire Osun state (latitude 7.5876° N and longitude 4.5624° E) is located in the tropical rainforest (average rainfall ranges from 1,125 mm in the derived savannah to 1,475 mm in the rainforest belt, with an annual temperature ranging from 27.2 °C in June to 39.0 °C in December of southwest Nigeria [43]. Therefore, water is collected in potholes and hollow objects around human dwellings and workplaces after rain (hence bushy surroundings and stagnant water around homes and workplaces). The majority of the participants in the study were Yoruba residents of Osogbo who sought medical attention at the four Primary Healthcare Centres (PHCs) chosen in the town. The Osun State University Health Research Ethics Committee (HREC) granted ethical approval for this study.

### Outcomes

The data were split into two categories, *Pf-positive* and *Pf*-negative, indicating those with and without malaria, respectively.

### Features

Participants were given a detailed explanation of the study protocol by the medical staff of the four Primary Health care facilities, and only those who provided written informed consent were recruited. Data on the socio-demographic behaviour, environment, and clinical characteristics of the subjects were gathered through questionnaires. Each participant’s body temperature, weight, and height were measured at appropriate facilities. Age less than 18 years and a lack of interest in participating in the study were requirements for exclusion. Information on age, sex, body weight, height, body mass index, body temperature, fever, diarrhoea, vomiting, headache, cough, sore throat, dizziness, muscle pain, presence of stagnant water at home, presence of stagnant water in the workplace, presence of bushes in the surroundings, and use of mosquito repellants were collected from the patients. This information was collected because these variables are commonly associated with malaria risk [44].

To ensure high quality of our data, we adhered to the specific guidelines and definitions of our methods. Fever was defined as an axillary temperature of ≥ 37.5 °C, in line with the World Health Organization’s standards [45]. The determination of bush proximity and density was achieved via GPS coordinates ‘close proximity’, defined as bushes within 100 m of a participant’s residence, and high bush density as > 50% area coverage [46, 47]. All *Pf* malaria diagnoses were RDT-confirmed, in line with the best practices of WHO [48].

### Statistical analysis

#### Patient baseline characteristics

Patients’ baseline characteristics were summarised using frequencies and proportions for categorical variables and medians and ranges for continuous variables. The characteristics were compared between *Pf*-positive and *Pf*-negative patients using the Wilcoxon Rank Sum test for continuous variables and Pearson’s chi-square test for categorical variables, with Yates’ continuity correction when appropriate. Indicators of significant associations between variables were set at P < 0.05.

#### Multivariable models development

Multivariable penalised logistic regression [49, 50], Bayesian generalised model [51, 52], and decision tree model [53–55] with nested cross-validation [56, 57] for parameter optimisation and wrapper-based sequential backward feature selection [58] were employed to determine the malaria type (*Pf*-positive or -negative). Randomly selected 80% of the samples (160 samples consisting of twenty-eight and one hundred thirty-two *Pf*-positive and Pf-negative samples, respectively) were used for the model training. The remaining 20% (40 samples consisting of seven and thirty-three *Pf*-positive and-negative samples, respectively) were used for testing.

#### Data scaling

Continuous variables in the training set were scaled to have a mean of 0 and standard deviations of 1 using the z-score algorithm, and the corresponding variables of the test set were mapped onto the space on the training set.

#### Nested cross-validation

Nested cross-validations (CVs) involving multiple layers of cross-validation (inner and outer folds) were performed on the training dataset to obtain reliable classification accuracy and avoid overfitting [56, 57]. The inner folds were used to optimise the model parameters and select useful feature subsets, and the performance of the best (inner) model was then evaluated in the outer fold. For the outer fold, we split the training dataset into a 30-fold cross-validation; one-fold was kept as a test set, while the remaining 29 folds (i.e. outer training fold) were, in turn, split in the inner fold into 20 stratified folds, 19 folds for model training, and the remaining fold for validation, to provide an unbiased evaluation of the model fit on the inner training set while tuning the model’s hyperparameters and selecting optimal features. The outer and inner folds were repeated 20 times to obtain a robust model. In addition, to address the imbalance in our dataset, we employed stratified k-folds in the outer and inner folds.

#### Optimal feature selection and hyperparameters

Feature selection was performed using sequential backward search selection (SBSS) for each inner training set [58]. The SBSS started with all features and dropped the non-informative features at each iteration, improving the model’s performance. This process was continued until no improvement was observed. Once the best combination of hyperparameters and feature subsets that maximised the performance metrics in the validation set was found, the model with the combination of hyperparameters and feature subsets was re-trained on the outer training set and tested on the test set kept out from the outer CV. The feature subsets from all outer folds were then combined using a voting strategy that retained features with more than 50% occurrences in all outer folds as informative; hence, they were chosen as the final feature subset [59]. The median of the best hyperparameters from the outer CV folds was used to fit the final model.

#### Performance evaluations

To generate summary performance estimates, we averaged the area under the curve (AUC) of the receiver operating characteristic (ROC) curve and other performance evaluations, such as sensitivity, specificity, positive predictive value (PPV), and negative predictive value (NPV) of the cross-validation [60, 61]. The sensitivity $\left(\frac{TP}{TP+FP}\right)$, specificity $\left(\frac{TN}{TN+FN}\right)$, PPV$\left(\frac{TP}{TP+FN}\right)$, and NPV $\left(\frac{TN}{TN+FP}\right)$, where TP, FP, TN, and FN are the numbers of true positives, false positives, true negatives, and false negatives, respectively, were calculated using the default cutoff value (0.5) for the *Pf*-positive or -negative classes for each model. We chose the model parameter values that led to the highest specificity values.

#### Package and software

All statistical analyses were performed using R. The machine- learning models were carried out using the Caret library (version 6.0.93). The receiver operating characteristic (ROC) curves of the models were drawn using the pROC library (version 1.18.0). We examined the association between model-selected predictors and the odds of malaria. The predictors and their corresponding adjusted odds ratios (AOR), confidence intervals (CI), and p-values are presented. The AOR estimates an increase in the odds of having malaria per unit increase in the predictor. The CI provides a range of values for the AOR, which are likely to contain the true value of the AOR with a 95% degree of confidence.

## Results

### Patient’s Characteristics

This training set included samples from 160 *Pf*-negative and *Pf*-positive patients (Table 1**)**. The median age of the patients was 41 years. Patients with *Pf* negativity tended to be older than those who tested positive for Pf (p = 0.025). In contrast, patients with *Pf* negativity were associated with lower body weight (p < 0.001), lower height (p = 0.03), and lower body mass index (p = 0.033) than those with *Pf* positivity. There was an association between *Pf* positivity and fever (p = 0.004), headache (p = 0.003), stagnant water at the workplace (p = 0.039), or bushes in the surroundings (p = 0.003). However, no association was observed between *Pf* positivity and sex, diarrhoea, cough, sore throat, dizziness, muscle pain, stagnant water at home, or mosquito repellant use. The baseline characteristics of patients in the training and test sets were similar (Table 2).

### Machine learning models for predicting malaria status

**Fig. 1:**
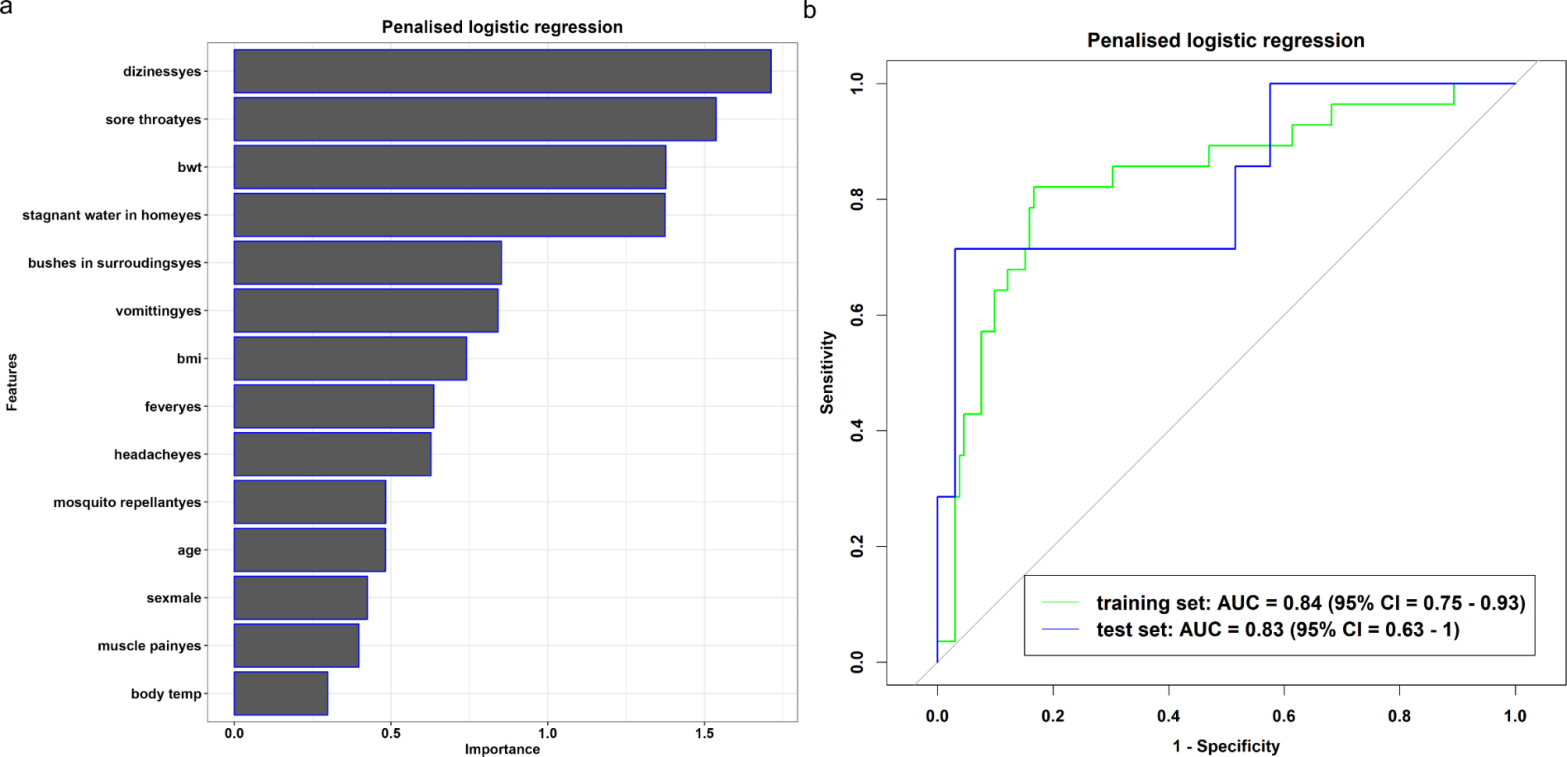
Features Important plot (**a**) and Roc curve (**b**) from multivariable penalised logistic regression model

**Fig. 2:**
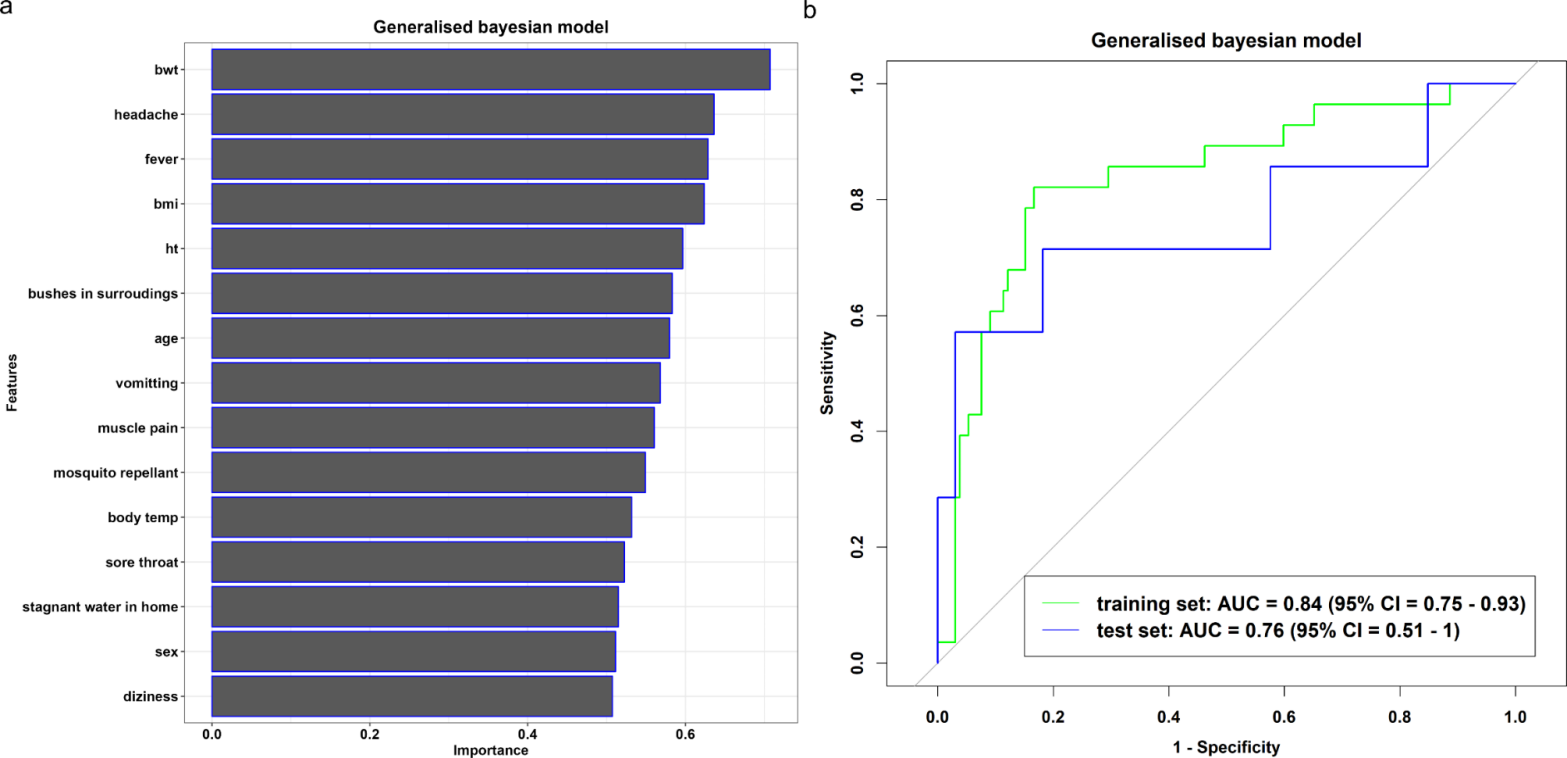
Features Important plot (**a**) and Roc curve (**b**) from Bayesian generalised model

**Fig. 3:**
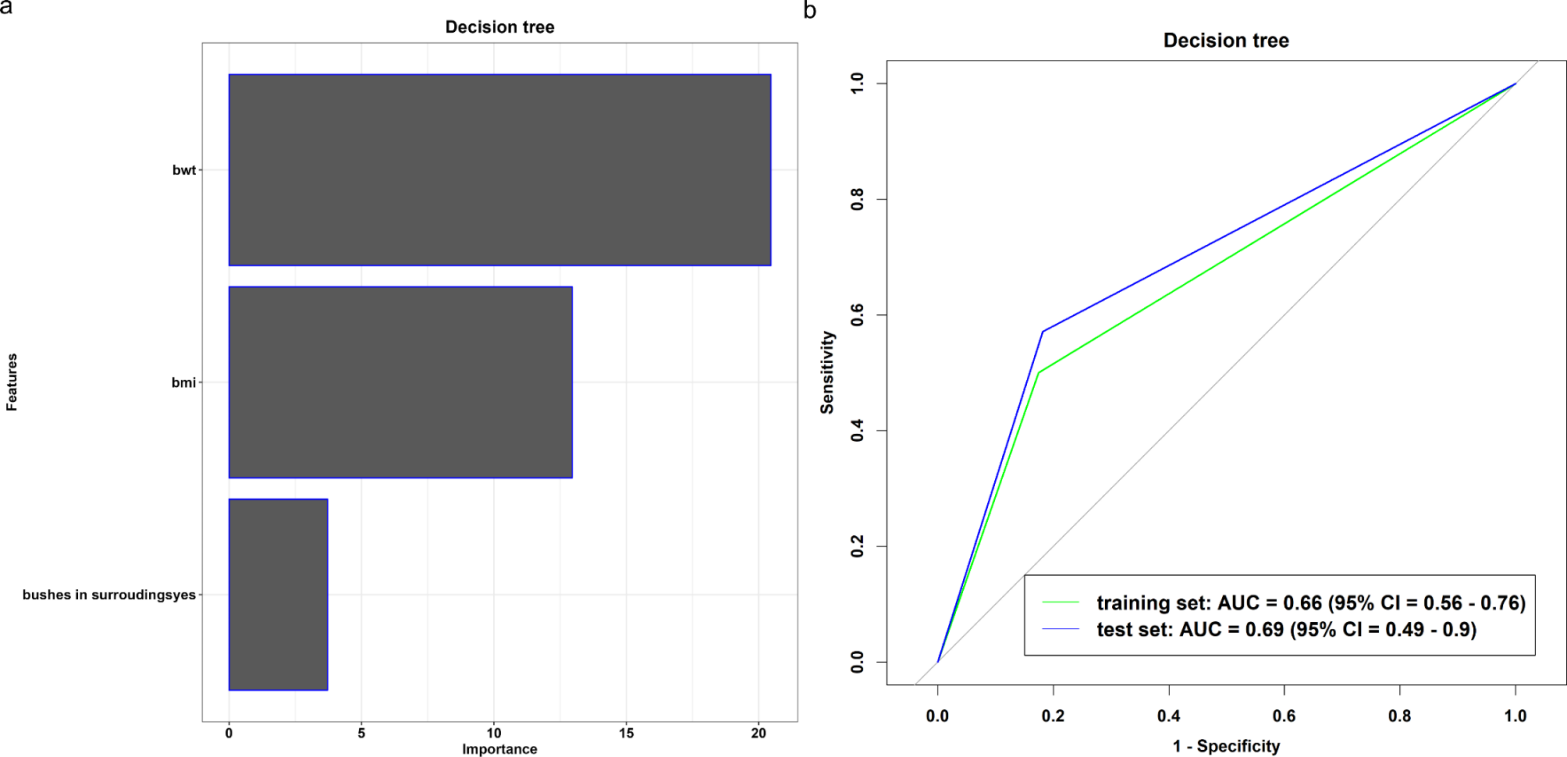
Features Important plot (**a**) and Roc curve (**b**) from decision tree model

We trained and tested each model and calculated the performance metrics for the training and test sets. The multivariable penalised logistic regression (Fig. 1a) and Bayesian generalised (Fig. 2a) models included patients’ body weight, headache, fever, body mass index, bushes in surroundings, age, vomiting, muscle pain, mosquito repellant, body temperature, sore throat, stagnant water at home, sex, and dizziness as the informative features, with training and test AUC (%) values: multivariable penalised logistic regression model (training: 84% vs. test: 83%; Fig. 1b), and Bayesian generalised model (training: 84% vs. test: 76%; Fig. 2b). The Bayesian generalised model also includes height as a part of the informative features. In contrast, the decision tree model included body weight, body mass index, and bushes in the surroundings as informative features (Fig. 3a), with AUC (%) values of 66% and 69% for the training and test datasets, respectively (Fig. 3b).

**Table 1:** Patients’ characteristics

Predictor	No (N = 132)	Yes (N = 28)	Total (N = 160)	p-value
**Age (years)**				0.025*
Median (Range)	42.000 (21.000, 72.000)	30.000 (23.000, 63.000)	41.000 (21.000, 72.000)	
**Sex**				1.000^†^
Female	111 (84.1%)	24 (85.7%)	135 (84.4%)	
Male	21 (15.9%)	4 (14.3%)	25 (15.6%)	
**Body Weight (Kg)**				< 0.001*
Median (Range)	59.000 (48.000, 86.000)	64.100 (49.900, 87.900)	59.650 (48.000, 87.900)	
**Height(m)**				0.030*
Median (Range)	1.690 (1.390, 1.820)	1.700 (1.580, 1.840)	1.700 (1.390, 1.840)	
**Body Mass Index (Kg/M** ^**2**^ **)**				0.033*
Median (Range)	21.080 (16.230, 29.070)	21.910 (18.550, 28.000)	21.135 (16.230, 29.070)	
**Body Temperature (°C)**				0.949*
Median (Range)	36.800 (34.000, 39.100)	36.750 (36.000, 39.000)	36.800 (34.000, 39.100)	
**Fever**				**0.004** ^†^
No	100 (75.8%)	13 (46.4%)	113 (70.6%)	
Yes	32 (24.2%)	15 (53.6%)	47 (29.4%)	
**Diarrhea**				0.780^†^
No	127 (96.2%)	26 (92.9%)	153 (95.6%)	
Yes	5 (3.8%)	2 (7.1%)	7 (4.4%)	
**Vomiting**				0.195^†^
No	125 (94.7%)	24 (85.7%)	149 (93.1%)	
Yes	7 (5.3%)	4 (14.3%)	11 (6.9%)	
**Headache**				**0.003** ^†^
No	94 (71.2%)	11 (39.3%)	105 (65.6%)	
Yes	38 (28.8%)	17 (60.7%)	55 (34.4%)	
**Cough**				1.000^†^
No	116 (87.9%)	24 (85.7%)	140 (87.5%)	
Yes	16 (12.1%)	4 (14.3%)	20 (12.5%)	
**Sore Throat**				0.654^†^
No	127 (96.2%)	28 (100.0%)	155 (96.9%)	
Yes	5 (3.8%)	0 (0.0%)	5 (3.1%)	
**Dizziness**				0.286^†^
No	130 (98.5%)	26 (92.9%)	156 (97.5%)	
Yes	2 (1.5%)	2 (7.1%)	4 (2.5%)	
**Muscle Pain**				0.375^†^
No	107 (81.1%)	20 (71.4%)	127 (79.4%)	
Yes	25 (18.9%)	8 (28.6%)	33 (20.6%)	
**Stagnant Water at Home**				0.239^†^
No	89 (67.4%)	15 (53.6%)	104 (65.0%)	
Yes	43 (32.6%)	13 (46.4%)	56 (35.0%)	
**Stagnant Water in the Workplace**				**0.039** ^†^
No	103 (78.0%)	16 (57.1%)	119 (74.4%)	
Yes	29 (22.0%)	12 (42.9%)	41 (25.6%)	
**Bushes in Surroundings**				**0.003** ^†^
No	86 (65.2%)	9 (32.1%)	95 (59.4%)	
Yes	46 (34.8%)	19 (67.9%)	65 (40.6%)	
**Use of Mosquito Repellent**				0.345^†^
No	55 (41.7%)	15 (53.6%)	70 (43.8%)	
Yes	77 (58.3%)	13 (46.4%)	90 (56.2%)	

*Wilcoxon test; ^†^ Pearson’s chi-square test with Yate’s correction was used for categorical variable

**Table 2:** Baseline characteristics of patients in the training and test sets

Predictor	Training (N = 160)	Test (N = 40)	Total (N = 200)	p-value
**Response**				1.000^†^
No	132 (8.5%)	33 (82.5%)	165 (82.5%)	
Yes	28 (17.5%)	7 (17.5%)	35 (17.5%)	
**Age (years)**				0.342*
Median (Range)	41.000 (21.000, 72.000)	38.500 (18.000, 64.000)	41.000 (18.000, 72.000)	
**Sex**				1.000^†^
Female	135 (84.4%)	34 (85.0%)	169 (84.5%)	
Male	25 (15.6%)	6 (15.0%)	31 (15.5%)	
**Body Weight(Kg)**				0.526*
Median (Range)	59.650 (48.000, 87.900)	58.300 (42.200, 76.100)	59.000 (42.200, 87.900)	
**Height(m)**				0.281*
Median (Range)	1.700 (1.390, 1.840)	1.675 (1.510, 1.800)	1.700 (1.390, 1.840)	
**Body Mass Index(Kg/M** ^**2**^ **)**				0.893*
Median (Range)	21.135 (16.230, 29.070)	21.330 (14.600, 28.130)	21.150 (14.600, 29.070)	
**Body Temperature(°C)**				0.874*
Median (Range)	36.800 (34.000, 39.100)	36.500 (35.700, 38.500)	36.800 (34.000, 39.100)	
**Fever**				0.969^†^
No	113 (70.6%)	29 (72.5%)	142 (71.0%)	
Yes	47 (29.4%)	11 (27.5%)	58 (29.0%)	
**Diarrhea**				0.928^†^
No	153 (95.6%)	39 (97.5%)	192 (96.0%)	
Yes	7 (4.4%)	1 (2.5%)	8 (4.0%)	
**Vomiting**				1.000^†^
No	149 (93.1%)	37 (92.5%)	186 (93.0%)	
Yes	11 (6.9%)	3 (7.5%)	14 (7.0%)	
**Headache**				0.287^†^
No	105 (65.6%)	22 (55.0%)	127 (63.5%)	
Yes	55 (34.4%)	18 (45.0%)	73 (36.5%)	
**Cough**				0.283^†^
No	140 (87.5%)	38 (95.0%)	178 (89.0%)	
Yes	20 (12.5%)	2 (5.0%)	22 (11.0%)	
**Sore Throat**				0.417^†^
No	155 (96.9%)	37 (92.5%)	192 (96.0%)	
Yes	5 (3.1%)	3 (7.5%)	8 (4.0%)	
**Dizziness**				1.000^†^
No	156 (97.5%)	39 (97.5%)	195 (97.5%)	
Yes	4 (2.5%)	1 (2.5%)	5 (2.5%)	
**Muscle Pain**				0.468^†^
No	127 (79.4%)	29 (72.5%)	156 (78.0%)	
Yes	33 (20.6%)	11 (27.5%)	44 (22.0%)	
**Stagnant Water at Home**				0.911^†^
No	104 (65.0%)	27 (67.5%)	131 (65.5%)	
Yes	56 (35.0%)	13 (32.5%)	69 (34.5%)	
**Stagnant Water in the Workplace**				0.108^†^
No	119 (74.4%)	24 (60.0%)	143 (71.5%)	
Yes	41 (25.6%)	16 (40.0%)	57 (28.5%)	
**Bushes In Surroundings**				0.638^†^
No	95 (59.4%)	26 (65.0%)	121 (60.5%)	
Yes	65 (40.6%)	14 (35.0%)	79 (39.5%)	
**Mosquito Repellent usage**				0.803^†^
No	70 (43.8%)	16 (40.0%)	86 (43.0%)	
Yes	90 (56 2%)	24 (60.0%)	114 (57.0%)	

^*^ Wilcoxon test; ^†^ Pearson’s chi-square test with Yate’s correction was used for categorical variables

**Table 3:** Performance of penalised logistic regression, Bayesian generalised, and decision tree models for training and test sets

Model	Data set	Sensitivity	Specificity	PPV	NPV	AUC (%)
penalised logistic regression	training	0.750	0.821	0.952	0.411	84
	Test	0.818	0.714	0.931	0.455	83
Bayesian generalised model	training	0.765	0.821	0.953	0.426	84
	Test	0.818	0.714	0.931	0.455	76
decision trees	training	0.826	0.500	0.886	0.378	66
	Test	0.818	0.818	0.900	0.400	69

The sensitivity, specificity, PPV, and NPV proportions from the models for the training and test datasets are presented in Table 3. The penalised logistic regression and Bayesian generalised models achieved similar sensitivity, specificity, PPV, and NPV values, outperforming the decision tree model. Comparisons of the AUC and other performance parameters revealed the advantage of the penalised regression model over other models in predicting the malaria class. The optimal parameters of the penalised logistic model were α = 0.025 and λ = 0.002.

**Table 4:** Adjusted odds ratios (AOR) from the multivariate penalised logistic regression model

Patients’ characteristics	AOR	95% CI	p-value
Age(years)	0.62	0.41–0.9	**0.012**
BMI(Kg/M^2^)	0.47	0.26–0.79	**0.006**
Body Temperature(°C)	1.40	0.99–1.91	0.054
Bushes in surroundings			
No	1		
Yes	2.60	1.3–4.66	**0.006**
Body Weight(Kg)	4.50	2.27–8.01	**< 0.0001**
Dizziness			
No	1		
Yes	0.30	0.05–0.94	**0.042**
Fever			
No	1		
Yes	2.10	0.88–4.24	0.099
Headache			
No	1		
Yes	2.07	0.95–3.95	0.068
Mosquito repellent usage			
No	1		
Yes	1.78	0.86–3.27	0.128
Muscle pain			
No	1		
Yes	1.63	0.66–3.39	0.333
Sex			
Female	1		
Male	0.72	0.24–1.71	0.373
Sore throat			
No	1		
Yes	0.26	0.1–0.55	**< 0.0001**
Stagnant water at home			
No	1		
Yes	0.26	0.11–0.53	**< 0.0001**
Vomiting			
No	1		
Yes	2.80	0.85–6.82	0.097

*Significant at p < 0.05; CI: confidence interval; AOR: adjusted odds ratio; reference categories (RC): AOR = 1. The RC for the bushes in the surroundings, dizziness, fever, headache, mosquito repellent, muscle pain, sore throat, stagnant water in the home, or vomiting is ‘No’, and the RC for sex is ‘Female’

### Relationships between patient features and malaria

Table 4 presents the adjusted odds ratios (AOR), AOR confidence intervals, and p-values of the predictors from the penalised logistic regression models. As shown in Table 4, increased odds of *Pf* antigen positivity (malaria) were associated with higher body weight (AOR = 4.50, 95% confidence interval (CI): 2.27 to 8.01, p < 0.0001) and high body temperature (AOR = 1.40, 95% CI: 0.99 to 1.91, p = 0.054). In contrast, decreased odds of Pf antigen positivity (malaria) were associated with age (AOR = 0.62, 95% CI: 0.41to 0.90, p = 0.012) and BMI (AOR = 0.47, 95% CI: 0.26 to 0.80, p = 0.006). Patients who had (or experienced) bushes in the surroundings (AOR = 2.60, 95% CI: 1.30 to 4.66, p = 0.006) or experienced fever (AOR = 2.10, 95% CI: 0.88 to 4.24, p = 0.099), headache (AOR = 2.07; 95% CI: 0.95 to 3.95, p = 0.068), muscle pain (AOR = 1.49; 95% CI: 0.66 to 3.39, p = 0.333), and vomiting (AOR = 2.32; 95% CI: 0.85 to 6.82, p = 0.097) were more likely to be positive for *the Pf* antigen test than those who did not have bushes in the surroundings, fever, headache, muscle pain, and vomiting, respectively. In contrast, male patients (AOR = 0.72; 95% CI: 0.24 to 1.71, p = 0.373), those who had (or experienced) dizziness (OR = 0.30; 95% CI: 0.05 to 0.94, p = 0.042), stagnant water at home (AOR = 0.26; 95% CI: 0.11 to 0.53, p < 0.0001), and sore throat (AOR = 0.26; 95% CI: 0.01 to 0.55, p < 0.0001) were less likely to be positive for *the Pf* antigen test (experience malaria) than female patients or those who did not have stagnant water at home, dizziness, or sore throat. Surprisingly, compared to those who did not use mosquito repellents, our data showed that patients who used mosquito repellents had higher odds of testing positive for *the Pf* antigen (developing malaria) (AOR = 1.78; 95% CI: 0.86 to 3.27, p = 0.128).

## Discussion

This study routinely collected sociodemographic, environmental, and clinical data to predict the incidence of *Pf* infections. Among the tested models, the penalised logistic regression model exhibited the best performance, with 84% and 83% training and test AUC accuracies, respectively, in predicting malaria status. Our results revealed associations between the presence of *Pf* (determined by RDT) and body mass index (BMI) (AOR = 0.47, 95% CI: 0.26 to 0.80, p-value = 0.006), body weight (AOR = 4.50, 95% CI: 2.27 to 8.01, p < 0.0001), dizziness (OR = 0.30; 95% CI: 0.05 to 0.94, p-value = 0.042), and sore throat (AOR = 0.26; 95% CI: 0.01to 0.55, p < 0.0001).

Body weight and BMI have been shown to affect the incidence of *Pf* malaria [62], which is consistent with our findings. Our results confirmed the need to consider patient BMI and weight when diagnosing *Pf* malaria, as these factors play significant roles in determining the presence of the disease. Although there have been a few reports of dizziness and sore throat as clinical signs of *Pf* malaria [63, 64], it is believed that changes in antioxidant marker levels and the status of several enzyme activities have been observed in patients with *Pf* malaria, suggesting that oxidative stress may play a significant role in malaria [65].

Our results also demonstrate a relationship between age and the prevalence of *Pf* infection, which is consistent with earlier research showing that younger people are more susceptible to malaria [66–69]. Thus, special interventions should be implemented for younger individuals because they are more vulnerable to *Pf* infections. In contrast, none of the other demographic features considered in this study was associated with the incidence of *Pf* infection.

Our findings also revealed associations between the positivity of *the Pf* antigen (malaria) and some environmental features, such as bushes in the surroundings (AOR = 2.60, 95% CI: 1.30 to 4.66, p = 0.006) and the presence of stagnant water (AOR = 0.26; 95% CI: 0.11 to 0.53, p < 0.0001). This study is in line with previous research demonstrating how environmental elements, such as vegetation and water bodies, might affect malaria transmission [70, 71]. Bushes can serve as breeding grounds for mosquitoes, which are the main carriers of malaria and can also offer shade and humidity, both of which are conducive to mosquito survival and reproduction. Thus, clearing bushes and other vegetation from the areas surrounding homes and communities can be a useful tactic for lowering the risk of malaria transmission. However, the use of mosquito repellents was not significantly associated with a reduced likelihood of malaria, which is not particularly surprising as reports have emerged that mosquitoes and other pests have become resistant to some routinely used repellents [72–74].

Unlike the work by [75, 76], which revealed associations between clinical symptoms, such as fever, vomiting, and headache, and the incidence of falciparum infection, it is interesting to note that our results revealed no significant associations between the occurrence of *Pf* and fever, vomiting, or headache, even though they all showed a high propensity for malaria. Our results showed that, although *Pf* typically causes symptoms such as fever, vomiting, and headache, these signs or symptoms are non-specific and can be mistaken for other illnesses [77].

In addition to the established factors previously identified in malaria prediction, our study introduces novel features that contribute to the accuracy and utility of the model. By incorporating environmental factors such as the presence of bushes in the surroundings and stagnant water in the home, the model acknowledges the role of the immediate environment in malaria transmission. This recognition of local ecological factors enhances the ability of the model to predict malaria occurrence in specific settings, thus tailoring the results to the unique risks faced by individuals in various regions. Furthermore, our model’s integration of these novel features highlights the importance of a holistic approach to understanding and addressing malaria transmission, which could ultimately lead to more effective intervention strategies.

Another innovative aspect of our study is the application of machine learning techniques to predict malaria occurrence using routinely collected data. By employing penalised logistic regression implemented under nested cross-validation with sequential backward feature selection, our model optimised its predictive power while minimising the risk of overfitting. This data-driven approach facilitates the identification of key predictors of malaria and provides a more precise prediction of malaria risk at an individual level. The use of machine-learning techniques in this context is not novel. Nevertheless, it demonstrates the potential of such models to enhance clinical decision making and resource allocation, particularly in resource-limited settings. This diligent application of machine learning has the potential to transform the way healthcare professionals approach malaria prevention and treatment, ultimately improving patient outcomes and the efficiency of the healthcare system.

Despite the relatively small sample size, which may limit the generalisability of our findings, we employed robust methodologies to ensure the reliability and validity of our results. Specifically, our use of nested cross-validation for hyperparameter search and sequential backward feature selection mitigated the risk of overfitting, which is a common pitfall in studies with limited data. Using these rigorous techniques, we optimised the extraction of meaningful insights from our dataset, thereby enhancing the reliability and validity of our findings. Consequently, while acknowledging the potential limitations imposed by the sample size, we maintain that our approach and analytical rigor provide a sound foundation for the results of this study.

Although our study relied on self-reported symptoms and environmental factors, we acknowledge that this method can introduce a recall bias or misclassification. However, we implemented stringent measures to mitigate these issues and to ensure the accuracy of our data. We addressed the recall bias using shorter recall periods. This approach minimised the chances of participants forgetting or misremembering the information, thereby increasing the reliability of their responses. We employed precise and accurate diagnostic techniques to minimise the risk of misclassification, particularly for malaria diagnoses. Routine rapid diagnostic testing, a highly sensitive and specific method for identifying Plasmodium species, was used for all the suspected malaria cases. This strategy greatly reduces the likelihood of misclassifying cases, and thus increases the accuracy of our data. Furthermore, we ensured that all the health workers involved in this study were highly experienced and thoroughly trained, which was critical to the robustness of our data collection process. Their expertise significantly minimised any potential errors that could have occurred during data collection. Despite these mitigation measures, we recognise that there is always potential for some level of bias in self-reported data. Future studies could consider incorporating additional methods to further reduce bias, such as triangulation of data through multiple data collection methods and sources or using more objective measurements where feasible. Despite these limitations, our study demonstrates the potential utility of machine learning models using sociodemographic, environmental, and clinical features to predict malaria occurrence.

## Conclusion

In conclusion, this study effectively employed penalised logistic regression to classify malaria types as *either* positive or negative. Our findings emphasise the significance of patient characteristics such as age, body weight, and symptoms in malaria diagnosis and management. In addition, stagnant water has been identified as a critical challenge in malaria control, necessitating interventions to address this issue. Implementing strategies such as regular cleaning and removal of stagnant water, community engagement, and promoting the use of insecticide-treated bed nets can help reduce the incidence of malaria. Educating people about risk factors and the need to seek medical attention for symptoms such as fever and headache can further contribute to the decline in malaria cases.

These findings enrich our understanding of the epidemiology of the disease and could potentially help prioritise preventive measures, particularly in resource-limited settings. However, it is crucial to reiterate that this predictive model is not intended to replace laboratory diagnosis. Instead, it was designed to augment them by providing an early indicator of potential disease incidence, particularly when resources for comprehensive laboratory testing are limited. Laboratory diagnosis remains the gold standard for identifying malarial infections, and our research aimed to complement this method by providing additional clues that could enhance its predictive power.

We focused on *Pf* because of its prevalence and severe impact in Nigeria and acknowledge that other malarial species are also relevant. Future studies should consider a more inclusive approach, investigate other Plasmodium species, and include more variables. This could further refine our understanding of the complex epidemiology of malaria in Nigeria and other similar contexts, ultimately leading to more effective strategies for malaria prediction and control. Our study underscores the need for and potential benefits of an integrated, multifaceted approach to predict and control malaria. Our findings support ongoing efforts to combat this disease, enhance the effectiveness of existing strategies, and offer new avenues for future research.

These findings may inform targeted interventions and contribute to the development of more accurate and efficient strategies for malaria prevention and control. In particular, this study may aid in clinical decision-making and resource allocation, particularly in resource-limited settings where traditional diagnostic methods are either unavailable or limited in accuracy. Finally, further research is needed to validate the model in larger and more diverse populations, and to assess its impact on patient outcomes and healthcare system efficiency.

## Data Availability

The datasets used in this study are available upon request from the corresponding author.
